# C-Phycocyanin Extract Modulates Thermogenic and Inflammatory Markers in Brown Adipose Tissue of High-Fat Diet-Fed Animals

**DOI:** 10.3390/molecules30122537

**Published:** 2025-06-10

**Authors:** Artur Francisco Silva-Neto, Julia Ferreira Rocha, Gustavo Oliveira Lima, Juliana Miki Oguma, Vivien Cayres Giarola Suannes Pucci, Yasmin Alaby Martins Ferreira, Maria Isabel Alonso-Vale, Claudia Maria Oller do Nascimento, Mônica Marques Telles, Anna Rafaela Cavalcante Braga, Luciana Chagas Caperuto, Lila Missae Oyama

**Affiliations:** 1 Department of Physiology, Escola Paulista de Medicina, Universidade Federal de São Paulo, São Paulo 04023-062, Brazil; artur.francisco@unifesp.br (A.F.S.-N.); jfrocha@unifesp.br (J.F.R.); gustavo.oliveira25@unifesp.br (G.O.L.); juliana.oguma@unifesp.br (J.M.O.); vivien.pucci@unifesp.br (V.C.G.S.P.); yasminalaby@hotmail.com (Y.A.M.F.); claudia.oller@unifesp.br (C.M.O.d.N.); mmtelles@unifesp.br (M.M.T.); 2 Department of Biological Sciences, Institute of Environmental Chemical and Pharmaceutical Sciences, Federal University of São Paulo, Diadema 09972-270, Brazil; alonso.vale@unifesp.br (M.I.A.-V.); caperuto@unifesp.br (L.C.C.); 3 Department of Chemical Engineering, Campus Diadema, Universidade Federal de São Paulo, Diadema 09972-270, Brazil; anna.braga@unifesp.br

**Keywords:** C-phycocyanin, high-fat diet, brown adipose tissue, metabolism, inflammatory markers, UCP-1, mice

## Abstract

C-phycocyanin (CPC), a bioactive compound derived from Spirulina, has been described as a molecule with antioxidant and anti-inflammatory properties. It has also been demonstrated that sustainably obtained CPC effectively inhibited body mass gain, regulated serum leptin and resistin levels, and prevented the onset of a pro-inflammatory state in Swiss mice fed a hyperlipidic diet. These results highlighted the anti-obesogenic potential of CPC. Brown adipose tissue (BAT) has been identified as a promising target in the treatment of obesity, playing a role in energy expenditure. In this study, Swiss mice fed a high-fat diet were supplemented with 500 mg/kg body weight of CPC daily for 12 and 16 weeks. BAT was collected, and Western blot and ELISA were performed. A reduction in pro-inflammatory cytokines, as well as a decrease in leptin levels was observed in the tissue, which was also associated with a reduction in BAT relative weight to body mass. Furthermore, CPC administration was able to modulate uncoupling protein 1 (UCP1) levels, which is crucial in the thermogenesis process. Therefore, this study demonstrated that supplementation with CPC reduces inflammatory cytokines associated with detrimental effects in the BAT, emerging as a tool in combating obesity and improving BAT function.

## 1. Introduction

Adipose tissue functions as an endocrine organ, significantly impacting overall body health. Its fat storage capacity, combined with its roles in energy homeostasis, insulin sensitivity, inflammation, and the secretion of various hormones and adipokines, makes it one of the most essential organs in studies involving obesity and metabolic diseases [1,2]. The heterogeneity of adipose tissue has also been highlighted, particularly related to the impact on health [3]. This tissue is classified into two types: white adipose tissue (WAT) and brown adipose tissue (BAT). While WAT primarily functions in energy storage, BAT is responsible for non-shivering thermogenesis [4].

BAT, primarily composed of brown adipocytes with large multilocular lipid droplets, maintains body temperature by dissipating energy as heat, a process mediated by uncoupling protein 1 (UCP1). In humans, BAT is found in regions such as the axillary, cervical, paravertebral, and supraclavicular areas, and its activity may be reduced in pathological conditions such as aging, diabetes, and obesity. Considered a potential target for weight loss, BAT promotes energy expenditure through the action of UCP1, which transports H+ ions across the mitochondrial membrane, generating heat instead of ATP [5,6]. Studies suggest that the lack of UCP1 in mice increases susceptibility to diet- and age-related obesity [7,8], which implies that BAT has a potential role against metabolic disorders and obesity [9,10].

In addition to the thermogenic function, BAT is a well-recognised endocrine tissue, releasing factors and hormones named BAT adipokines or batokines. Cytokines like IL-6, leptin, angiogenesis inducer protein as VEGF, and the browning inducer factor like FGF21 showed autocrine, paracrine, and endocrine actions. By endocrine actions, some batokines can target tissues such as white adipose tissue, liver, pancreas, heart and bone, and central neural system, integrating and affecting the systemic metabolism [11].

With the global rise in obesity, there is a growing search for nutritional solutions, including the use of cyanobacteria such as Spirulina, which has anti-inflammatory, antioxidant, and hypolipidemic properties [12,13]. Recently, our group demonstrated that the bioactive compound of Spirulina, C-phycocyanin (CPC), sustainably obtained, effectively inhibited body mass gain, regulated serum leptin and resistin levels, and prevented the increase of a pro-inflammatory state in Swiss mice fed a hyperlipidic and hypercaloric diet [14,15]. Seo et al. [16] used an ethanolic extract of Spirulina maxima as a treatment for ICR mice fed a high-fat diet for six weeks and observed lower expression of adipogenic proteins and higher expression of thermogenic factors in WAT, demonstrating the extract’s ability to inhibit adipogenesis and activate the thermogenic expression program.

However, the literature shows a lack of data regarding the effects of bioactive compound CPC on BAT inflammatory and thermogenesis markers. Therefore, this study analysed the benefits presented by these nutraceuticals on brown adipose tissue, focusing on factors involved in thermogenesis and inflammatory processes in a mouse model fed a hyperlipidic and hypercaloric diet. The green aspect of the C-phycocyanin extraction process is worth mentioning, using minimal chemical components that injure the environment.

## 2. Results

### Characterization of the Model and the Effect of CFC Extract on Thermogenesis Protein Expression and Inflammatory Processes in BAT

In regard to TNF-α concentration in BAT, there was no difference between the ND and HFD groups after 12 weeks of the protocol; however, after 16 weeks of the protocol, the HFD group showed a significant increase compared to the ND group. As a result of the increased inflammatory response in BAT, the TNF-α/IL-10 ratio showed significant differences in both protocols, with a notable increase in IL-10 in the HFD group after 16 weeks. These results occurred even though the BAT relative weight was not statistically different between these groups in both treatments. Additionally, MCP-1 exhibited a significant increase after 16 weeks, indicating that the hyperlipidic and hypercaloric diet induced a pro-inflammatory process in BAT. Regarding the model’s validation, the adipokine leptin showed a significant increase in both protocols in the HFD group. In contrast, resistin increased only after 16 weeks of diet. Regarding the amount of UCP-1 present in BAT, a decrease was observed in the HFD group after 16 weeks. IL-6, STAT3, PPARγ, and PGC1 showed no significant differences in both treatments. These results are shown in Table 1.

**Figure 1:**
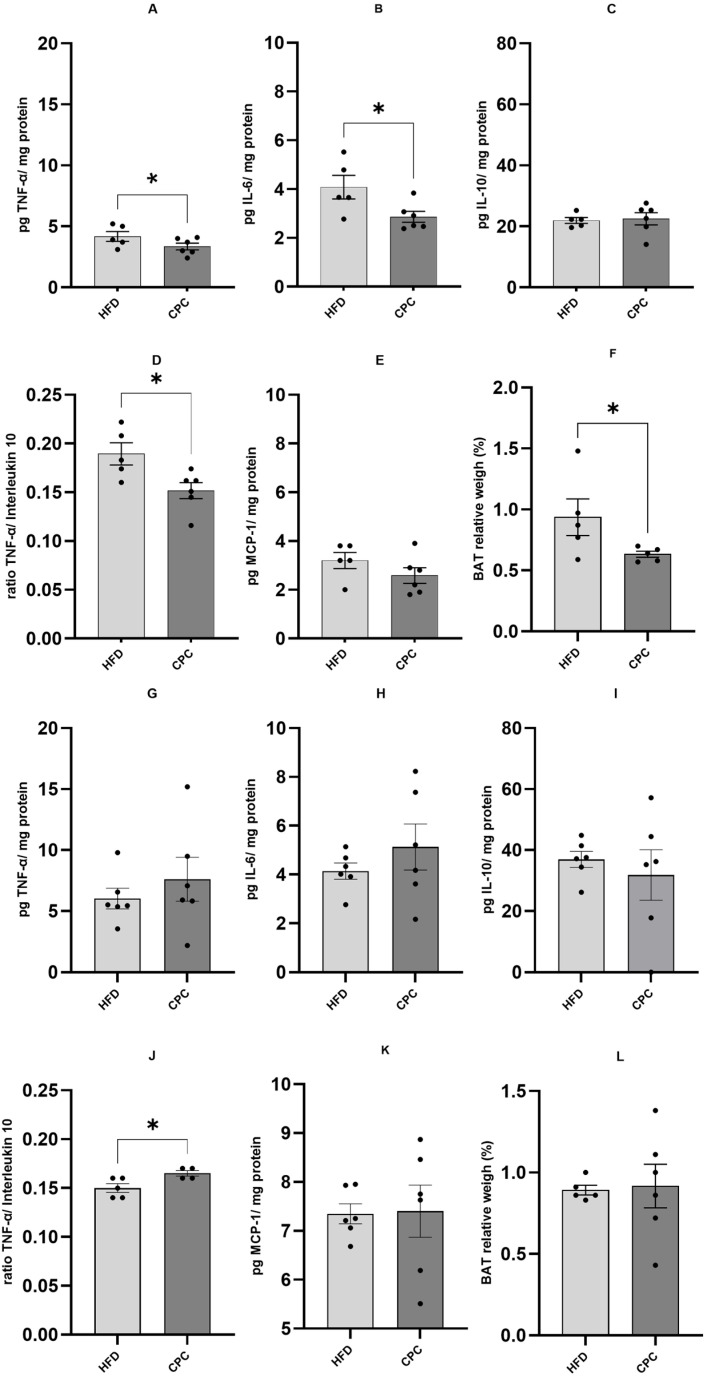
Cytokine concentration in brown adipose tissue, in picogram of cytokine per mg of protein, and brown adipose tissue relative weight. Comparison between HFD and CPC groups. Figures (**A**–**F**) represent the 12-week treatment (*n* = 5 in the HFD group and *n* = 6 in the CPC group), while (**G**–**L**) represent the 16-week treatment (*n* = 6 both groups). (**A**,**G**): TNF-α; (**B**,**H**): IL-6; (**C**,**I**): IL-10; (**D**,**J**): TNF-α/IL-10 ratio; (**E**,**K**): MCP-1; (**F**,**L**): BAT relative weight. * different from the HFD group (*p*< 0.05). The bar on the graph represents the standard error of the mean.

The administration of C-phycocyanin alongside a hyperlipidic and hypercaloric diet for 12 weeks resulted in significant decrease (*p* < 0.05) in the concentrations of pro-inflammatory cytokines such as TNF-α and IL-6, compared to the HFD group. When evaluating the TNF-α/IL-10 ratio, a significant reduction (*p* < 0.05) in the pro-inflammatory process was observed in the CPC group compared to the HFD group. On the other hand, no such differences were observed after 16 weeks of hyperlipidic diet and C-phycocyanin administration, with only an increase in the inflammatory process in the TNF-α/IL-10 ratio in the CPC group compared to the HFD group. The BAT relative weight was lower in the CPC group compared to the HFD group in the 12-week protocol, but this same result was not observed in the 16-week protocol. No significant difference was observed for IL-10 and MCP-1 in both treatments. These results are shown in Figure 1.

**Figure 2:**
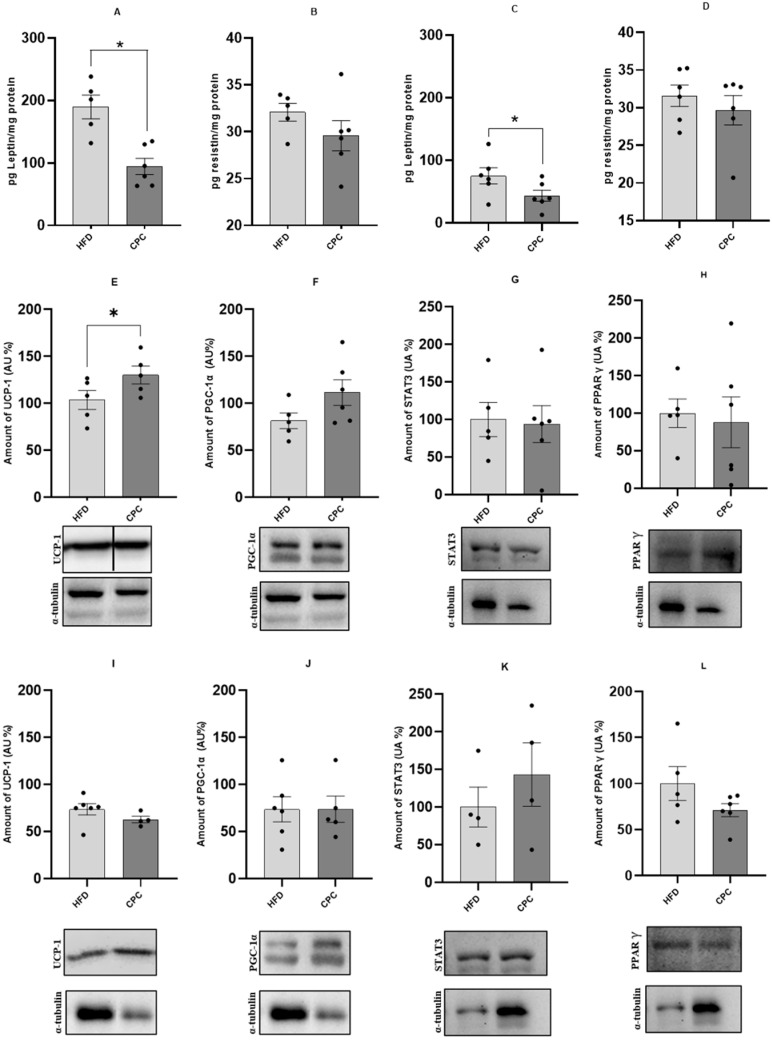
Concentration of adipokines leptin and resistin in brown adipose tissue, in picograms per milligram of protein. Comparison between the HFD and CPC groups. (**A**,**B**): Leptin and resistin at 12-week treatment (*n* = 5 in the HFD group and *n* = 6 in the CPC group), respectively; (**C**,**D**): Leptin and resistin at 16-week treatment (*n* = 6 both groups), respectively. Figures (**E**–**L**) represent the intensity of each protein band analysed in brown adipose tissue and the respective housekeeping (alpha tubulin) at 12 and 16-week treatment. Figure (**E**) at H: UCP-1, PGC-1α, STAT3 and PPARγ at 12-week treatment (*n* = 5–6 in HFD and CPC group), respectively, while figure (**I**) at L represents the same proteins at 16-week of treatment (*n* = 4–6 in HFD and CPC group). * different from the HFD group (*p* < 0.05). The bar on the graph represents the standard error of the mean. Note: The UCP-1 band shown in Figure (**E**) was cropped due to the presence of additional samples on the Western blot gel that were not included in this study.

Regarding leptin, the administration of C-phycocyanin in the HFD group reduced adipokine levels in both treatments (*p* < 0.05), but no differences were observed for resistin levels. The protein expression of UCP-1 in the CPC group showed an increase at the end of the 12-week protocol compared to the HFD group (*p* < 0.05), but after 16 weeks, the same result was not observed. There were no differences in the amounts of PGC-1α, STAT3, and PPARγ when comparing these two groups in both treatments. These results are shown in Figure 2.

## 3. Discussion

The increase in the thermogenesis process can be a coadjuvant tool to help normalize metabolism in some metabolic diseases like obesity. Since the C-phycocyanin intake has demonstrated an anti-obesity effect, this study analysed the thermogenesis and inflammatory markers in the brown adipose tissue of mice treated with a hyperlipidic diet associated with C-phycocyanin supplementation. Although heat production was not analysed, the reduction in body weight in CPC is associated with thermogenesis marker levels and depends on the duration of the treatment.

BAT is a specialized tissue that regulates metabolism, increasing energy expenditure through heat production. Nowadays, it is well established that the BAT also has an endocrine function, which operates by releasing factors named batokines, including cytokines and hormones, with autocrine, paracrine, and endocrine action [11].

The activation of BAT thermogenesis can be a therapeutic application to control weight in metabolic diseases like obesity. In this sense, the anti-obesogenic effect of C-phycocyanin can be attributed partially to the restoration of thermogenesis, demonstrated by an increase in UCP-1 protein content, although this effect is time-dependent. Notably, the DIO mouse showed a decrease in BAT UCP-1 protein content compared to the normolipidic diet treatment. UCP1 regulates the mitochondrial membrane potential, supporting heat generation, as demonstrated in the review by Ferreira et al. [17].

Although numerous studies have demonstrated that an obesogenic diet, high in fat and sugar, increases BAT weight and upregulates UCP-1 content, long-term exposure to a hyperlipidic and hypercaloric diet, as supported in this study, leads to a decline in the UCP-1 content. This reduction is associated with an elevated pro-inflammatory state within the tissue. It is well-established that the chronic subclinical inflammation characteristic of obesity can induce a pro-inflammatory environment in BAT, which negatively impacts its thermogenic activity and energy expenditure [18]. These findings suggest that BAT initially attempts to enhance energy expenditure in response to dietary challenges. However, its functionality becomes compromised over time, partly due to the development of pro-inflammatory conditions.

Another important observation to consider when assessing the pro-inflammatory state induced by hyperlipidic and hypercaloric diet in BAT is an increase in tissue leptin content. The high level of leptin associated with increased white adipose depots is well documented [19]. However, the specific augment on BAT local concentration has no particular indication. Mori et al. [20] stated that the increase in leptin and decrease in UCP-1 gene expression in BAT could indicate a whitening process.

Leptin is involved in many physiological processes, such as appetite control, immunity, and energy homeostasis, and in our model is upregulated by HFD. The hyperleptinemia state can exacerbate metabolic dysfunction [21]. A slight decrease in plasma levels enhances leptin sensitivity, suggesting a potential new approach for obesity treatment [21]. Our previous study [14] demonstrated that CPC modulated serum leptin levels, influencing body mass gain over 12 and 16 weeks of treatment. The reduction in this hormone in BAT of mice treated with hyperlipidic and hypercaloric diet, associated with the decrease in pro-inflammatory batokines resulting from CPC administration, highlights the potential of this approach for obesity treatment.

Of note is that the death of brown adipocytes leads to decreased UCP-1 content. Kotzbeck et al. [22] showed that the inflammatory state associated with obesity promotes a whitening process in BAT adipocytes, increasing in size and heading towards death. Seo et al. [16] had already demonstrated in the white adipose tissue of obese mice that a Spirulina extract can positively regulate browning markers. We have shown that C-phycocyanin obtained from Spirulina has this property independently of the browning process, positively regulating the amount of UCP-1 in BAT and reducing its inflammatory state caused by the diet.

Restoration of these molecular markers may constitute a significant advancement in the development of therapeutic strategies for obesity and associated metabolic disorders. Treatment with CPC has demonstrated potential as a natural therapeutic agent, effectively normalizing UCP-1 expression and facilitating a transition towards an anti-inflammatory physiological state.

It is essential to emphasize that the main objective of this study was to demonstrate the deleterious impact of a hyperlipidic and hypercaloric diet on key metabolic markers in BAT. Rather than assessing the direct functional output of BAT, such as thermogenic capacity, this study highlights that a reduction in UCP-1, a critical protein for BAT’s thermogenic function, alongside elevated levels of pro-inflammatory markers such as TNF-α and leptin, characterizes the tissue’s dysfunction. Our findings show that C-phycocyanin can positively regulate UCP-1 even in animals fed with a hyperlipidic and hypercaloric diet, while also reducing its inflammatory state. These results are accompanied by a reduction in the local concentration of leptin, demonstrating that this bioactive compound may effectively combat obesity.

## 4. Materials and Methods

### 4.1. Preparation of C-Phycocyanin Extract

The C-phycocyanin extract was obtained using water as a solvent, and the extraction process has been previously published [15]. Briefly, Spirulina (*Arthrospira platensis* strain) was kindly provided by Fazenda Tamanduá^®^ (Santa Teresinha, Paraíba, Brazil). For the extraction process, Spirulina biomass was removed from the freezer (−38 °C) 1 h prior to the procedure and processed in an analytical mill (IKA A11 Basic, Guangzhou, China) to separate the particles using a mesh granulometry method (0.106 mm/Tyler mesh). Distilled water was added to the Spirulina sample, which was maintained at room temperature, followed by centrifugation for 120 min at 10,000× *g* (NT 816, Nova Analítica^®^ Diadema, São Paulo, Brazil), and the supernatant was separated from the remaining biomass. After the recovery of the liquid fraction of the extract, the sample was filtered using a vacuum system and a 0.22 μm hydrophilic syringe filter. For the precipitation process, the sample was fractionated with (NH_4_)_2_SO_4_, according to the methodology of Amarante et al. [23] and Silva et al. [24], to partially purify the C-phycocyanin and obtain a food-grade extract (purity > 0.7). For purity analysis, the C-phycocyanin sample was measured using a spectrophotometer at absorbances A28/A620/A652. Preliminary results using this C-phycocyanin extract in other tissues have been previously published [14,15].

### 4.2. Animals

All procedures were performed according to protocols approved by the Experimental Research Committee of the Federal University of São Paulo (CEUA no. 8626090821). Briefly, thirty-five non-isogenic male Swiss mice after completing 60 days of life were divided into three experimental groups each, one protocol lasting 12 weeks and the other lasting 16 weeks, namely: normolipidic diet + saline solution (ND); high-fat diet + saline solution (HFD); and high-fat diet + C-phycocyanin extract (CPC). Diets followed the recommendations of the American Institute of Nutrition (AIN-93) [25] and were prepared in our laboratory following protocols adapted from AIN-93 previously published [26,27]. Animals were kept in a room under controlled conditions of light cycle (12 h light/12 h dark) and temperature (24° ± 1 °C) and received food and water ad libitum. The ND group was fed a control diet, and the HFD and CPC groups were fed a hyperlipidic and hypercaloric diet in both treatments (Table 2). The ND and HFD groups received 0.9% saline daily by gavage, while the CFC group received C-phycocyanin at a concentration of 500 mgkg^−1^ body weight by gavage. The chosen dose was based on a study conducted by Brito et al. [28] in a dose–response evaluation, using 50, 150, and 500 mgkg^−1^ body weight per day, where they found effects of reduction in oxidative stress and inflammation markers at the dose of 500 mg/kg/day. The animals observed no signs of constipation or discomfort during the entire treatment. At the end of the experimental period, the mice were fasted for 12 h, anesthetized (80 mg/kg body weight of ketamine and 10 mg/kg body weight of xylazine), and euthanized by cardiac puncture. BAT was collected, weighed, and stored at −80 °C.

### 4.3. Protein Extraction for Western Blotting Method

BAT samples were homogenized in a lysis buffer (100 mM Trizma base pH 7.5), 10 mM EDTA, 10% SDS, 100 mM sodium fluoride, 10 mM sodium pyrophosphate, 10 mM sodium orthovanadate, 2 mM PMSF (Phenylmethylsulfonyl Fluoride), and aprotinin 0.1 mg/mL. The homogenate was centrifuged at 16,000× *g* for 45 min at 4 °C, and the supernatant was collected. The total protein concentration was determined using a spectrophotometer (NanoDrop™ One/OneC Microvolume UV–Vis Spectrophotometer, Thermo Scientific™, Wilmington, NC, USA). Proteins from the lysates were electrophoretically separated on a 10% SDS polyacrylamide gel and transferred to a nitrocellulose membrane. Membranes were blocked in 1% bovine serum albumin overnight at room temperature and then incubated overnight with the following primary antibody: UCP-1 (Abcam, Cambridge, UK—ab23841), PGC-1α (Thermo Fisher Scientific, Waltham, MA, USA—PA5-38021), STAT3 (Cell Signaling Technology, Danvers, MA, USA—#9139) and PPARγ (Abcam—ab45036). The α-tubulin antibody (Santa Cruz Biotechnology, Dallas, TX, USA—sc-58667) was used for further normalization and membrane analyses. The membranes were incubated with distinct primary antibodies targeting proteins of different molecular weights. In these cases, the same loading control from the corresponding membrane was used for the quantification analyses. Membranes were washed in TBS-T 1× and incubated with peroxidase-associated secondary antibodies for one hour. Bands were visualized with chemiluminescence scanned on UVITEC 4.7 Cambridge (Cambridge, UK) after adding ECL reagent (GE Healthcare Bio-Sciences AB, Little Chalfont, UK). The intensity of the bands was quantified using the Scion Image^®^ software alpha 4.0.3.2 (National Institute of Health, Bethesda, MD, USA).

### 4.4. Cytokines Concentration for ELISA Method

Samples of BAT were homogenized with a lysis buffer previously described (see Section 4.3) and centrifuged at 16,000× *g* for 45 min at 4 °C. The supernatant was used to measure pro-inflammatory cytokines (TNF-α, IL-6, IL-1β, and MCP-1), anti-inflammatory cytokines (IL-10) concentrations, and leptin and resistin concentrations, using commercial ELISA kits (R&D Systems^®^, Minneapolis, MN, USA). The procedures followed the manufacturer’s recommendations.

### 4.5. Statistical Analysis

GraphPad Prism version 8 (Dotmatics, Boston, MA, USA) was used for the statistical analysis. Data were subjected to Shapiro–Wilk (normality) and Kolmogorov–Smirnov (Equality) tests. To define the effects of the hyperlipidic and hypercaloric diet, a *t*-test was performed between the ND and HFD groups. To evaluate the effects of C-phycocyanin on the diet, we compared the HFD group with the CPC group using the same test. The significance level used in all analyses was set at less than 5% (*p* < 0.05).

## 5. Conclusions

This study demonstrated that supplementation with C-phycocyanin in animals fed a hyperlipidic and hypercaloric diet can regulate the amount of UCP-1 present in BAT. Furthermore, the bioactive compound was shown to reduce inflammatory batokines associated with detrimental effects in the tissue and decrease leptin concentration. These findings indicate the potential of C-phycocyanin as a promising agent for combating obesity and enhancing brown adipose tissue functionality.

## Figures and Tables

**Table 1:** Comparing normal and high-fat diet-treated animals for 12 or 16 weeks for BAT parameters.

Variable	ND 12-Week	HFD 12-Week	ND 16-Week	HFD 16-Week
BAT relative weight	0.70 ± 0.07	0.93 ± 0.14	1.10 ± 0.11	0.89 ± 0.02
UCP-1	100 ± 4.67	103.6 ± 10.13	100 ± 14.30	73.61 ± 5.99 *
PGC1-α	100 ± 10.99	81.50 ± 8.35	100 ± 22.39	73.53 ± 13.32
TNF-α	3.46 ± 0.19	4.18 ± 0.39	4.00 ± 0.41	6.02 ± 0.85 *
STAT3	100 ± 21.18	70.05 ± 11.90	100 ± 19.95	81.54 ± 21.62
PPARγ	100 ± 21.54	71.48 ± 5.44	100 ± 5.76	113.8 ± 8.32
IL-6	3.60 ± 0.22	4.07 ± 0.48	3.51 ± 0.27	4.13 ± 0.33
IL10	21.93 ± 1.26	21.94 ± 0.97	29.02 ± 2.13	36.92 ± 2.61 *
TNFα/IL-10	0.15 ± 0.01	0.18 ± 0.01 *	0.13 ± 0.005	0.15 ± 0.004 *
MCP-1	2.80 ± 0.29	3.20 ± 0.32	6.33 ± 0.31	7.34 ± 0.20 *
Leptin	93.79 ± 27.08	189.8 ± 19.01 *	24.71 ± 3.46	75.20 ± 12.89 *
Resistin	30.12 ± 1.15	32.09 ± 0.95	27.75 ± 0.97	31.61 ± 1.43 *

ND: normolipidemic group (*n* = 5–6 in both treatments); HFD: hyperlipidic group (*n* = 5 in 12-week and *n* = 6 in 16-week); * different from the ND group (*p* < 0.05). Data are presented as mean ± standard error.

**Table 2:** Composition of the normolipidic diet (ND) and high-fat diet (HFD).

Ingredients	ND (g/kg Diet)	HFD (g/kg Diet)
Maize starch	720.7	450
Sugar	–	150
Casein	140	180
Soy oil	40	40
Lard	–	180
Cellulose	50	–
Mix of vitamins/minerals	10/35	10/35
L-cystine/choline bitartrate/BHT	1.8/2.5/0.008	1.8/2.5/0.008
Caloric value (kcal/kg)	3802.8	5100
Carbohydrate (% energy)	75.81%	47.06%
Protein (% energy)	14.73%	14.12%
Lipids (% energy)	9.47%	38.82%

## Data Availability

All data are available and can be requested from the correspondence author.

## Abbreviations

The following abbreviations are used in this manuscript:

BAT	Brown adipose tissue
WAT	White adipose tissue
UCP1	Uncoupling Protein 1
CPC	C-phycocyanin
IL	Interleukine
TNF-α	Tumor necrosis factor alpha
MCP-1	Monocyte Chemotactic Protein 1
PGC-1α	Peroxisome proliferator-activated receptor gamma coactivator 1-alpha
STAT3	Signal transducer and activator of transcription 3
PPARγ	Peroxisome proliferator-activated receptor gamma
ND	Normolipidic diet
HFD	High-fat diet
